# *Chlamydia pneumoniae* Clinical Isolate from Gingival Crevicular Fluid: A Potential Atherogenic Strain

**DOI:** 10.3389/fcimb.2015.00086

**Published:** 2015-11-25

**Authors:** Simone Filardo, Marisa Di Pietro, Giovanna Schiavoni, Gianluca Minniti, Emanuela Ortolani, Silvio Romano, Rosa Sessa

**Affiliations:** ^1^Department of Public Health and Infectious Diseases, “Sapienza” UniversityRome, Italy; ^2^General Dentistry and Emergency Care Unit, George Eastman Dental HospitalRome, Italy; ^3^Department of Life, Health & Environmental Sciences, University of L'AquilaL'Aquila, Italy

**Keywords:** *Chlamydia pneumonia*e, atherosclerotic cardiovascular diseases, chronic periodontitis, gingival crevicular fluid, interleukin-1β, interleukin-6

## Abstract

*Chlamydia pneumoniae* has been associated to atherosclerotic cardiovascular diseases. The aim of our study was to characterize, for the first time, a *C. pneumoniae* strain isolated from the gingival crevicular fluid of a patient with chronic periodontitis, described as a risk factor for cardiovascular diseases. *C. pneumoniae* isolate was characterized and compared to the respiratory AR-39 strain by VD4-*ompA* genotyping and by investigating the intracellular growth in epithelial and macrophage cell lines and its ability to induce macrophage-derived foam cells. Inflammatory cytokine levels were determined in the gingival crevicular fluid sample. *C. pneumoniae* isolate showed a 99% similarity with the AR-39 strain in the VD4-*ompA* gene sequence and shared a comparable growth kinetic in epithelial cells and macrophages, as evidenced by the infectious progeny and by the number of chlamydial genomic copies. *C. pneumoniae* isolate significantly increased the number of foam cells as compared to uninfected and LDL-treated macrophages (45 vs. 6%, *P* = 0.0065) and to the AR-39 strain (45 vs. 30%, *P* = 0.0065). Significantly increased levels of interleukin 1-β (2.1 ± 0.3 pg/μL) and interleukin 6 (0.6 ± 0.08 pg/μL) were found. Our results suggest that *C. pneumoniae* may harbor inside oral cavity and potentially be atherogenic, even though further studies will be needed to clarify the involvement of *C. pneumoniae* in chronic periodontitis as a risk factor for cardiovascular diseases.

## Introduction

*Chlamydia pneumoniae* (*C. pneumoniae*) is an intracellular obligate pathogen with a unique developmental cycle consisting of an extracellular infectious form (elementary body, EB) responsible for transmitting the infection and an intracellular replicative form (reticulate body, RB) responsible for growth and multiplication within host cell. A chlamydial persistent form has recently been described as a strategy to escape the host immune response, resulting in a long-term infection. Chlamydial persistent forms are viable but not infectious, as indicated by continuous genomic DNA replication and reduced production of EBs (Schoborg, 2011; Di Pietro et al., 2012).

In the last two decades, *C. pneumoniae*, a common cause of respiratory tract infections, has been widely associated with atherosclerosis, a chronic inflammatory disease of the vascular wall, by seroepidemiological studies, *C. pneumoniae* DNA detection in the atherosclerotic plaque and by the isolation of viable bacteria from the atheroma (Campbell and Rosenfeld, 2015). *C. pneumoniae* persistent form is believed to mediate the chronic inflammatory state, as evidenced by an increased production of pro-inflammatory cytokines, contributing to chronic inflammatory diseases such as cardiovascular diseases (Atik et al., 2010; Campbell et al., 2010; Schoborg, 2011).

*C. pneumoniae* is presumed to play a role in the pathogenesis of atherosclerosis for its ability to disseminate systemically from the lungs through peripheral blood mononuclear cells (PBMCs) and to localize in extra-pulmonary sites such as the vascular wall (Moazed et al., 1998; Watson and Alp, 2008). Once inside the vascular tissue, *C. pneumoniae* has been shown to induce endothelial dysfunction and low density lipoprotein (LDL) oxidation, resulting in the accelerated uptake of cholesterol by macrophages, and the subsequent foam cell formation, considered as the hallmark of early atherosclerotic lesions (Kalayoglu et al., 1999; He et al., 2009). *C. pneumoniae* has also been demonstrated to contribute to platelet adhesion and aggregation and smooth muscle cell proliferation and migration, all events responsible for progression and rupture of vascular lesion and, hence, for acute cardiovascular events (Di Pietro et al., 2013a; Chatzidimitriou et al., 2014).

Several risk factors for atherosclerosis have been identified, including traditional (smoking, hypertension, hyperlipidemia, and diabetes) and non-traditional factors (inflammation, oxidative stress, and infections; Balagopal et al., 2011; Di Pietro et al., 2013a). Recently, chronic periodontitis has also been described as a predisposing factor for atherosclerotic cardiovascular diseases (Kholy et al., 2015). Indeed, it has been reported that patients with chronic periodontitis have a 19% greater risk of atherosclerotic cardiovascular disease than healthy individuals (Kurita-Ochiai and Yamamoto, 2014).

Chronic periodontitis, an inflammatory disease of periodontal tissue resulting from oral infections, is characterized by the formation of a periodontal pocket where gingival crevicular fluid drastically increases in response to oral pathogens, resulting in local severe inflammation, and destruction of the periodontium. The major pathogen responsible for periodontitis is *Porphyromonas gingivalis*, known to cause gingival ulceration, epithelial barrier destruction and, hence, to translocate into the systemic circulation, contributing to endothelial dysfunction, first step of the atherosclerotic process (Kebschull et al., 2010).

*C. pneumoniae* has also been suggested to harbor into the oral cavity, since it has been shown to infect gingival fibroblasts, resident cells of the periodontium, and to increase the inflammatory state underlying chronic periodontitis (Rizzo et al., 2008a,b).

Following the destruction of the periodontium, *C. pneumoniae* may enter the bloodstream, migrate into the vascular wall and induce the production of circulating cytokines and chemokines, contributing directly or indirectly to atherogenesis and, hence, to atherosclerotic cardiovascular diseases.

Here we isolated and characterized, for the first time, a *C. pneumoniae* strain from the gingival crevicular fluid of a patient with chronic periodontitis via specific gene typing. Furthermore, the phenotypical characteristics of *C. pneumoniae* clinical isolate were also investigated, followed by a comparative analysis to the reference strain AR-39.

## Materials and methods

### Gingival crevicular fluid and peripheral blood samples

Specimens of gingival crevicular fluid and peripheral blood were obtained from a 61-year old man suffering from chronic periodontitis diagnosed in accordance to the criteria proposed by the 1999 International World Workshop for a Classification of Periodontal Disease and Conditions (four sites with probing pocket depth ≥ 6 mm and attachment loss ≥ 4 mm; Armitage, 1999).

The patient, referred to George Eastman Dental Hospital for dental or periodontal treatment, received neither professional cleaning nor antibiotic treatment in the last 6 months.

He was free from any cardiovascular and respiratory disease; he was an ex-smoker and had hypertension, diabetes and a family history of cardiovascular diseases.

A total of 16 gingival crevicular fluid samples, eight from the deepest periodontal pocket and eight from four healthy periodontal sites, were collected by using sterile paper strips (#30PerioPaper). Before sampling, the sites for gingival crevicular fluid collection were gently air-dried, isolated from saliva with cotton rolls and the supra-gingival plaque was removed; then, paper strips were inserted in the sites for 10 s. Four paper strips were pooled in a sterile microcentrifuge tube containing sucrose-phosphate-glutamate buffer (SPG, 0.2 mol/L sucrose, 3.8 mmol/L KH_2_PO_4_, 6.7 mmol/L Na_2_HPO_4_, 5.5 mmol/L glutamic acid at pH 7.4) and four paper strips in a sterile microcentrifuge tube containing phosphate buffered saline (PBS) for culture and real-time PCR assays respectively. The ultimate volume of the pooled gingival crevicular fluid samples from the diseased and healthy sites were about 4 and 1.3 μL, respectively. Strips contaminated with blood were discarded and a new site was selected.

All gingival crevicular fluid samples were stored at −80°C until further processing.

Peripheral blood specimen (5 mL) was used for the isolation of PBMCs through Ficoll–Hypaque density gradient centrifugation.

Written informed consent was obtained from the patient and the study was approved by the International Review Board of “Sapienza” University of Rome.

### *C. pneumoniae* DNA detection by real-time PCR

Genomic DNA was extracted from 200 μL of diluted gingival crevicular fluid sample and from 10^6^ PBMCs using the QIAamp DNA Mini Kit (Qiagen) according to the manufacturer's instructions. *C. pneumoniae* AR-39 infected HEp-2 epithelial cells (ATCC CCL 23) and mock-infected HEp-2 cells were used as positive and negative extraction controls respectively.

Detection and quantification of *C. pneumoniae* DNA were performed by real-time PCR targeting MOMP gene using the Primerdesign™ genesig® Kit according to the manufacturer's instructions. Each PCR-run contained the PCR-negative control (ultrapure water PCR grade) and positive and negative extraction controls. DNA sample, positive and negative extraction controls were analyzed in triplicate. The sample was considered positive if all of three assay results were positive in the replicate test.

### *C. pneumoniae* detection by direct fluorescence assay

The gingival crevicular fluid samples from the diseased and healthy periodontal sites were centrifuged on cytoslide by cytospin for 5 min at 400 rpm (Thermo Scientific Cytospin 4, Thermo Fisher Scientific). Then, the cells were fixed with methanol, and stained with fluorescein isothiocyanate (FITC)-conjugated monoclonal antibody against chlamydial lipopolysaccharide (Pathfinder Chlamydia Culture Confirmation System, Biorad). The presence of *C. pneumoniae* inclusions was determined by fluorescence microscope (400x magnification).

### Isolation of *C. pneumoniae*

Clinical isolate was recovered in HEp-2 cells as previously described by Dowell et al. (2001). Briefly, 200 μL of diluted gingival crevicular fluid sample was inoculated on HEp-2 cells grown in 24-well plates in Dulbecco's Modified Eagle's medium high glucose (DMEM, Euroclone, Italy) supplemented with 10% (v/v) fetal calf serum (FCS), 100 μg/mL streptomycin, 250 μg/mL amphotericin B, 1% non-essential amino acids, and 1% sodium pyruvate (complete growth medium). Infected HEp-2 cells were centrifuged for 1 h at 1250 g at 37°C, and then incubated at 37°C and 5% CO_2_ for 3 h. Infected cell monolayers were then overlaid with complete growth medium supplemented with 2% FCS and 1 μg/mL cycloheximide, and incubated at 37°C and 5% CO_2_ for 72 h. Then, infected cells were further passaged to fresh HEp2 cell monolayers. In order to evaluate the development of chlamydial inclusions, infected HEp-2 cells, after each growth cycle, were stained with FITC-conjugated monoclonal antibody against chlamydial lipopolysaccharide. *C. pneumoniae* inclusion forming units (IFUs) were determined by counting all microscope fields using a fluorescence microscope (400x magnification).

### *C. pneumoniae* VD4-*ompA* genotyping

The VD4 region within *ompA* gene was selected as a target for the typing of *C. pneumoniae* clinical isolate. A nested PCR assay targeting a 366 bp fragment of the *C. pneumoniae ompA* gene was performed as previously described by Bodetti et al. (2002). The *ompA* PCR products were separated by gel electrophoresis and excised from the gel using a Gel Extraction Kit (Qiagen). Sense and anti-sense strands were sequenced with an automated DNA sequencer using the BigDye Terminator v1.1 Cycle sequencing Kit (Applied Biosystems). The sequence obtained was analyzed using BLAST 2 (http://www.ncbi.nlm.nih.gov/blast) and compared to *C. pneumoniae* VD4 sequences available in the GenBank database including AR-39 and TW-183 strains (GeneBank accession numbers AE017159, AE002161, AE001363, BA000008, AF131889, AY426606, AY426607).

### Growth characteristics of *C. pneumoniae* clinical isolate

HEp-2 and J774A.1 macrophage cells (ATCC TIB-67) (1 × 10^5^ cells/well) were infected with *C. pneumoniae* clinical isolate or AR-39 strain at a multiplicity of infection (MOI) of 1.0 by centrifugation for 1 h at 1250 g at 37°C. After the removal of chlamydial inoculum, infected cells were incubated in DMEM supplemented with 2% FCS and 1 μg/mL cycloheximide at 37°C and 5% CO_2_ for up to 72 h.

At 24, 48, and 72 h post-infection, the infectious progeny and genomic copy number of *C. pneumoniae* were detected.

The infectious progeny was determined by the development of chlamydial inclusions after passage to fresh HEp-2 cell monolayers. Briefly, cell monolayers were disrupted and passaged to fresh HEp-2 cell monolayers grown on glass coverslips in 24 well-plates. After 72 h of incubation at 37°C and 5% CO_2_, the number of IFUs/mL was determined by counting all microscope fields using a fluorescence microscope (400x magnification).

The genomic copy number of *C. pneumoniae* DNA was detected by real-time PCR using the Primerdesign™ genesig® Kit as above described.

### Foam cell analysis

J774A.1 macrophages (1.0 × 10^5^ cells/well) were infected with *C. pneumoniae* clinical isolate and AR-39 strain at a MOI of 2.0 as above described. Next, infected cells were washed with PBS and cultured for 2 days in the presence or absence of human LDL (100 μg/mL; Sigma-Aldrich, St. Louis, MO). Foam cell formation and intracellular cholesterol levels were determined as previously described (Di Pietro et al., 2013b).

### Cytokine analysis

Interleukin (IL)-1β and IL-6 concentrations were measured in gingival crevicular fluid samples by quantikine HS human enzyme-linked immunosorbent assay (ELISA) according to manufacturer's instructions (R&D systems, Europe). The detection limits for IL-1β and IL-6 were 0.14 and 0.11 pg/mL respectively, and, if the cytokine concentrations were below the limits of detection, a value of 0 was scored.

### Statistical analysis

Statistical analysis was performed by using SPSS 13.0 (SPSS Inc. Chicago, IL, USA). Data were expressed as mean ± standard deviation of three replicates from three independent experiments. Comparison of means was performed by using a two-tailed Student's *t*-test. For the foam cell formation and the cholesteryl ester to total cholesterol ratio, the Kruskal-Wallis H test and the Mann-Whitney U test with the Bonferroni's correction were used. *P* ≤ 0.05 was considered statistically significant.

## Results

### *C. pneumoniae* clinical isolate

*C. pneumoniae* DNA was detected in gingival crevicular fluid collected from diseased periodontal site by quantitative real-time PCR targeting MOMP gene. As compared to the calibration curve derived from serial dilutions of *C. pneumoniae* DNA, the average quantitative-PCR cycle threshold values in gingival crevicular fluid corresponded to the presence of 142,950 copies of *C. pneumoniae* DNA per microliter of sample.

**Figure 1:**
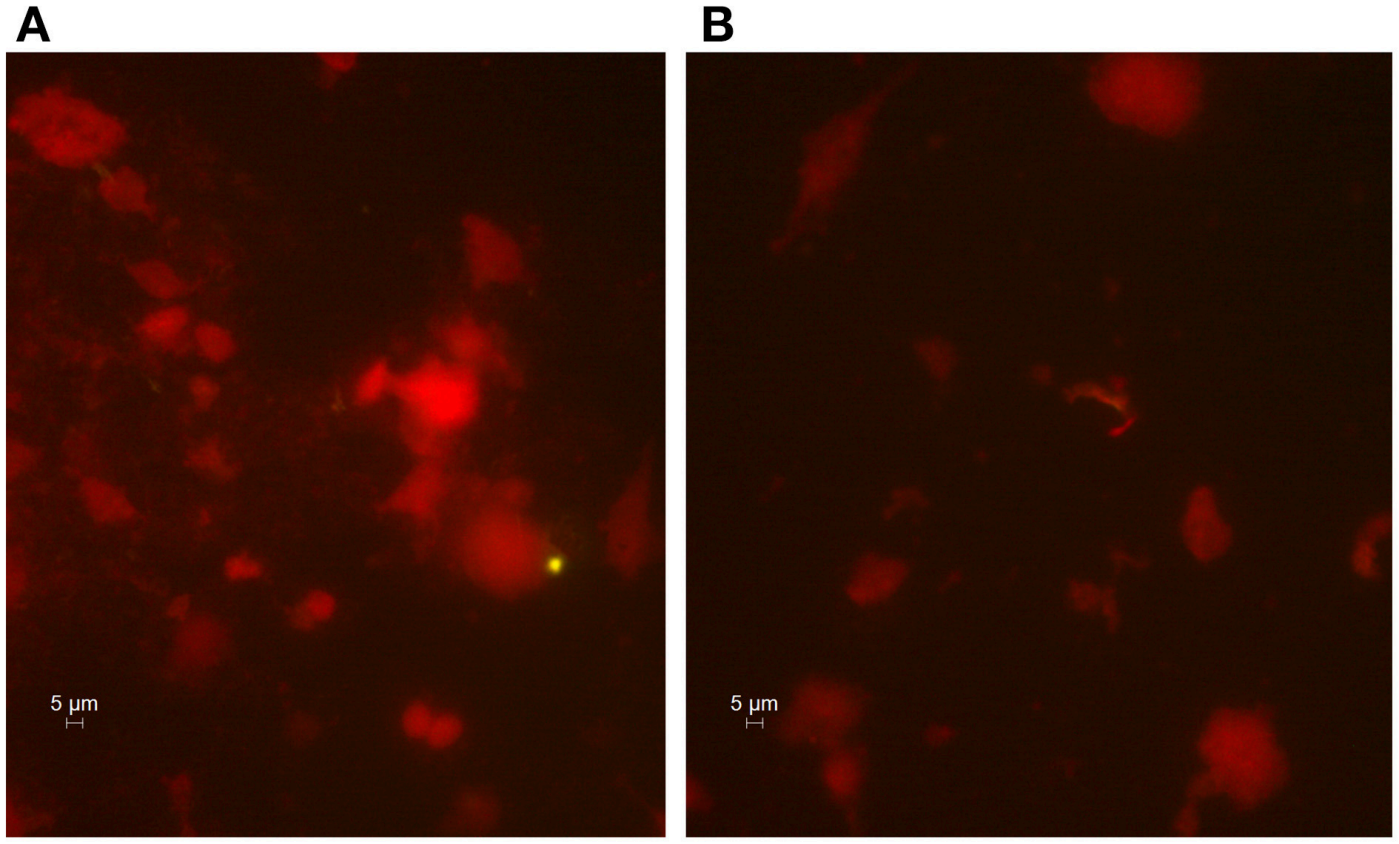
**Immunohistological staining of cells from the gingival crevicular fluid**. Presence of *C. pneumoniae* in the diseased periodontal site **(A)** and absence in the healthy site **(B)**. The gingival crevicular fluid samples were stained, and FITC-marked chlamydial inclusions (green) were visualized by fluorescence microscope (400x magnification).

In addition, the gingival crevicular fluid from the diseased periodontal site was positive to *C. pneumoniae* when subjected to direct fluorescence assay as evidenced by the presence of intracellular Chlamydia (Figure 1).

Conversely, no *C. pneumoniae* DNA, as well as chlamydial inclusions, was detected in gingival crevicular fluid obtained from healthy periodontal site.

Next, the crevicular gingival fluid sample positive to *C. pneumoniae* was subjected to culture in HEp-2 cells. An average of 50 chlamydial inclusions was detected in gingival crevicular fluid after three growth cycles of 3 days. The presence of *C. pneumoniae* in HEp-2 cell monolayers was confirmed by real-time PCR assay targeting *C. pneumoniae* MOMP gene.

Further characterization of clinical isolate was performed via *C. pneumoniae* VD4-*ompA* genotyping. As a result of this analysis, the VD4 segment of *ompA* gene sequence from *C. pneumoniae* clinical isolate was found to be 99% identical to the human *C. pneumoniae* AR-39 and TW-183 sequences.

### *C. pneumoniae* DNA detection in PBMCs

No *C. pneumoniae* DNA was detected in PBMCs from the patient with chronic periodontitis, despite the quantitative real-time PCR revealed the presence of *C. pneumoniae* DNA in the gingival crevicular fluid.

### Growth characteristics of *C. pneumoniae* clinical isolate

**Figure 2:**
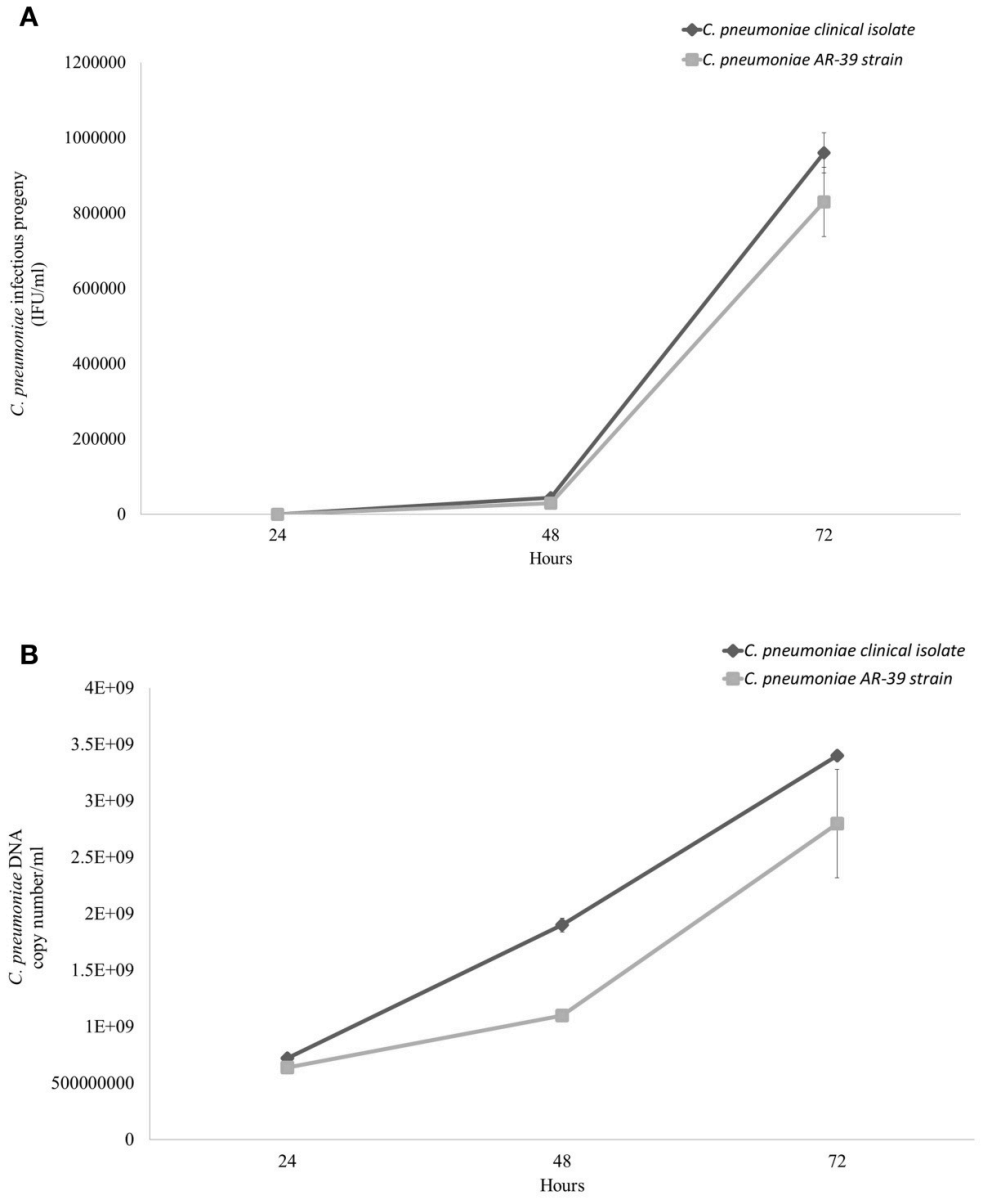
**Comparative growth of *C. pneumoniae* clinical isolate and AR-39 strain in HEp-2 epithelial cells**. HEp-2 cells were infected with *C. pneumoniae* clinical isolate and AR-39 strain at a MOI of 1.0. At 24, 48, and 72 h post-infection, **(A)** infectious progeny and **(B)** genomic copy number of both strains were assessed as described in Materials and Methods.

**Figure 3:**
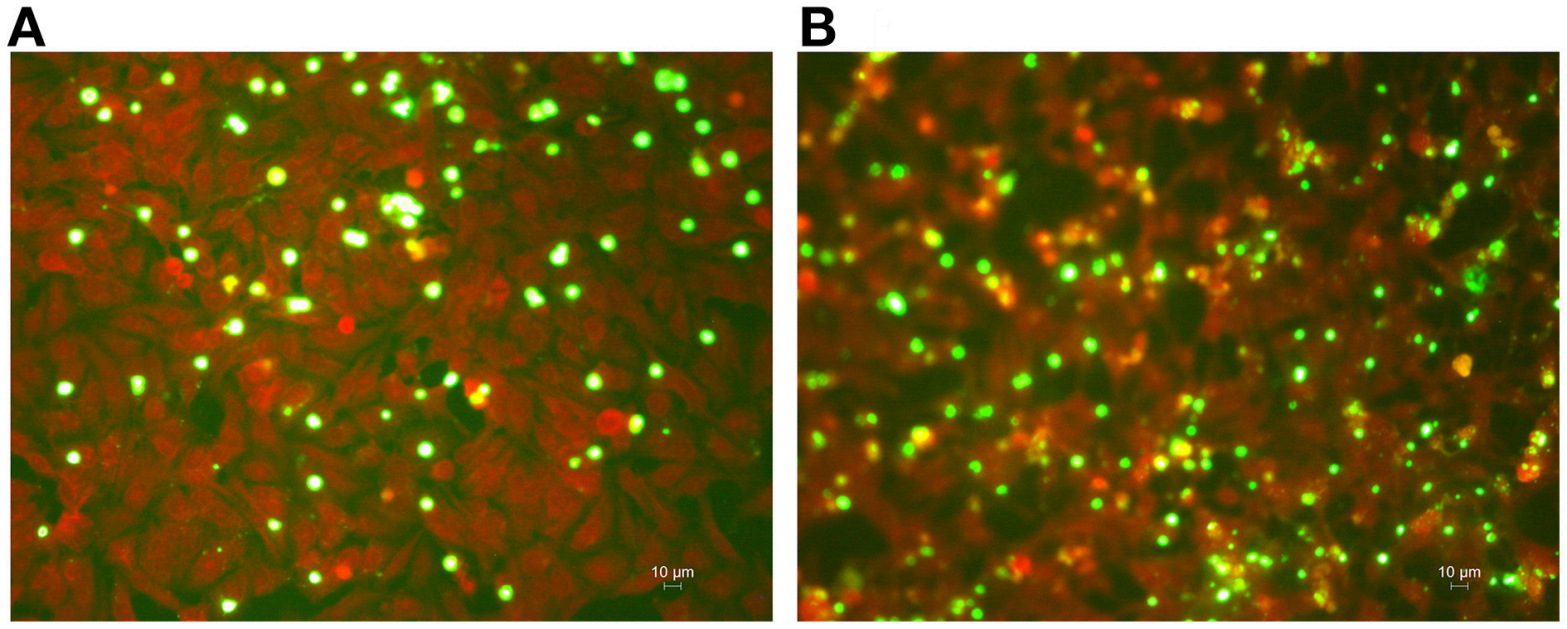
**Immunohistological staining of HEp-2 cell monolayers infected with *C. pneumoniae* clinical isolate and AR-39 strain**. HEp-2 cells were infected with **(A)** *C. pneumoniae* clinical isolate and **(B)** AR-39 strain (MOI 1.0). After 72 h of incubation, HEp-2 cell monolayers were fixed, stained, and FITC-marked chlamydial inclusions (green) were visualized by fluorescence microscope (400x magnification).

The intracellular growth of *C. pneumoniae* clinical isolate was first analyzed in HEp-2 epithelial cells. Specifically, the infectious progeny as well as the genomic copy number of *C. pneumoniae* were detected at 24, 48, and 72 h post-infection. As shown in Figure 2A, *C. pneumoniae* clinical isolate and AR-39 strain displayed a similar kinetic growth with increases of IFUs of three orders of magnitude for both strains. Although *C. pneumoniae* clinical isolate showed a higher growth titer than AR-39 strain, there was no statistically significant difference in the infectious progeny between the two strains at 24, 48, and 72 h post-infection. No significant difference in inclusion morphology and size was also observed between *C. pneumoniae* clinical isolate and AR-39 strain (Figure 3). The quantitative detection of MOMP gene of *C. pneumoniae* by real-time PCR of infected HEp-2 cells further revealed a similar intracellular growth of the clinical isolate to the AR-39 strain (Figure 2B).

**Figure 4:**
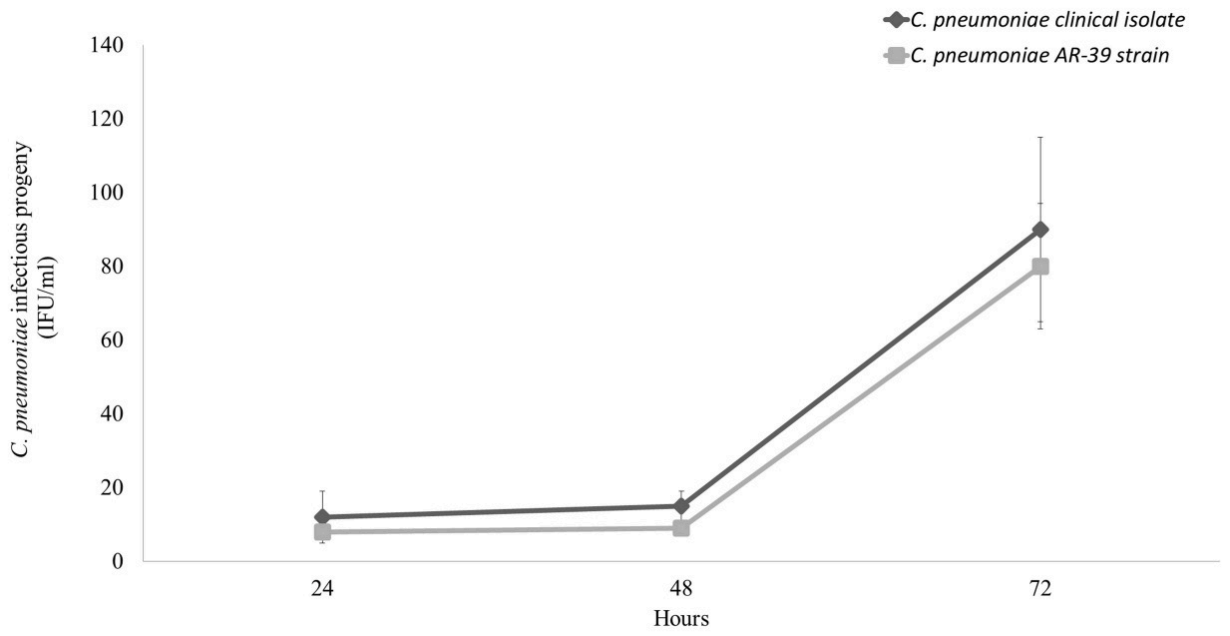
**Comparative growth of *C. pneumoniae* clinical isolate and AR-39 strain in macrophage cell line**. Macrophages were infected with *C. pneumoniae* clinical isolate and AR-39 strain at a MOI of 1.0. At 24, 48, and 72 h post-infection, infectious progeny was assessed as described in Materials and Methods.

Next, we examined the intracellular growth of *C. pneumoniae* clinical isolate in a macrophage cell line since the latter has been widely used to investigate the aspects of chlamydial host cell infection related to the atherosclerotic process. *C. pneumoniae* clinical isolate did not replicate efficiently in macrophages as evidenced by a low number of infectious EBs detected at 24, 48, and 72 h post-infection as compared to that detected in HEp-2 cells (*P* < 0.00001). Specifically, the number of infectious EBs remained unaltered at 24 and 48 h while it increased significantly at 72 h post-infection (*P* < 0.01). However, the levels of infectious EBs produced by *C. pneumoniae* clinical isolate were similar to those produced by the AR-39 strain (Figure 4).

### *C. pneumoniae* clinical isolate-induced foam cell formation

In order to evaluate the pro-atherogenic effects of the *C. pneumoniae* strain isolated from the gingival crevicular fluid, we determined the number of macrophage-derived foam cells as well as the ratio of intracellular cholesteryl ester to total cholesterol after 48 h incubation in the presence of LDL. A Kruskal-Wallis H test showed a statistically significant difference in the foam cell formation and the ratio of cholesteryl ester to total cholesterol between the different groups [uninfected macrophages, macrophages infected with *C. pneumoniae* clinical isolate and macrophages infected with the AR-39 strain, χ^2^(2) = 15.16, *P* = 0.0005].

**Figure 5:**
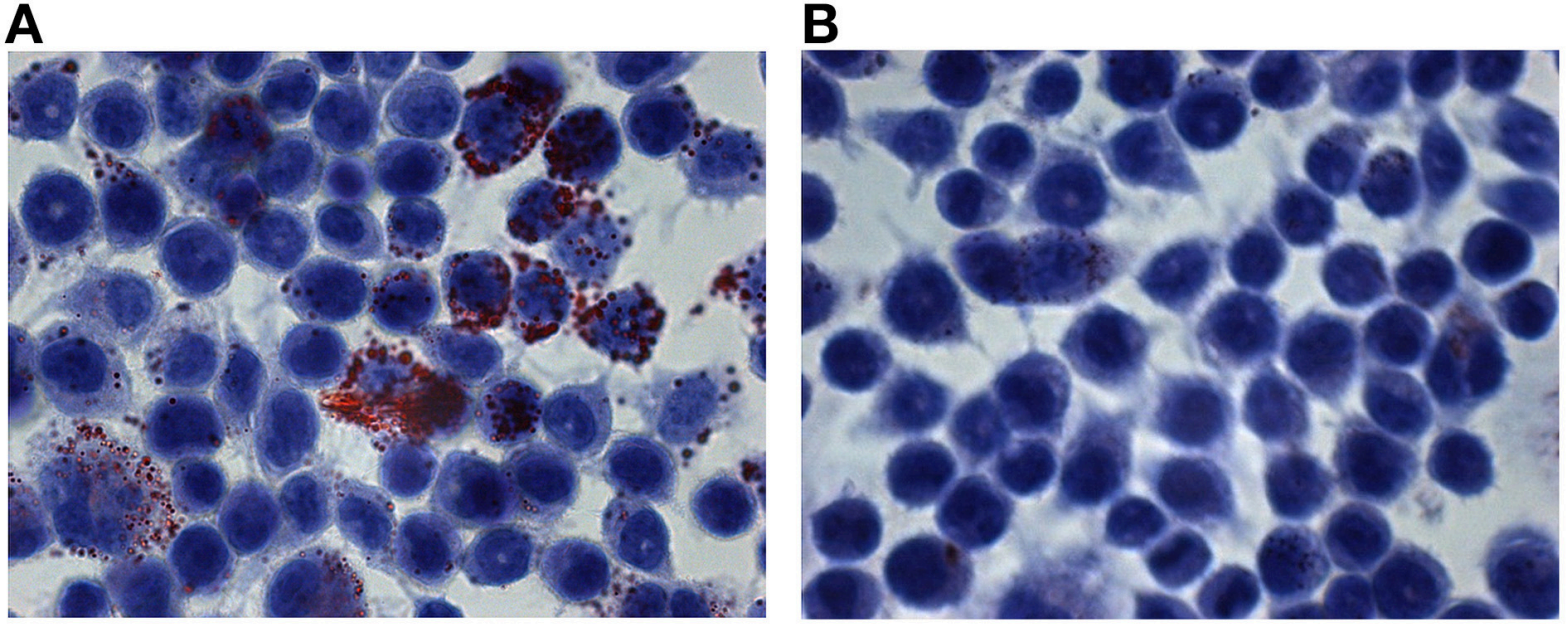
**Atherogenic features of *C. pneumoniae* clinical isolate**. Macrophages were infected with *C. pneumoniae* clinical isolate (MOI 2.0) and incubated in the **(A)** presence or **(B)** absence of LDL. After 48 h, the foam cells were identified by using Oil-Red O staining. Micrograph images show Oil-Red O stained macrophages with large amounts of intracellular lipid droplets.

**Figure 6:**
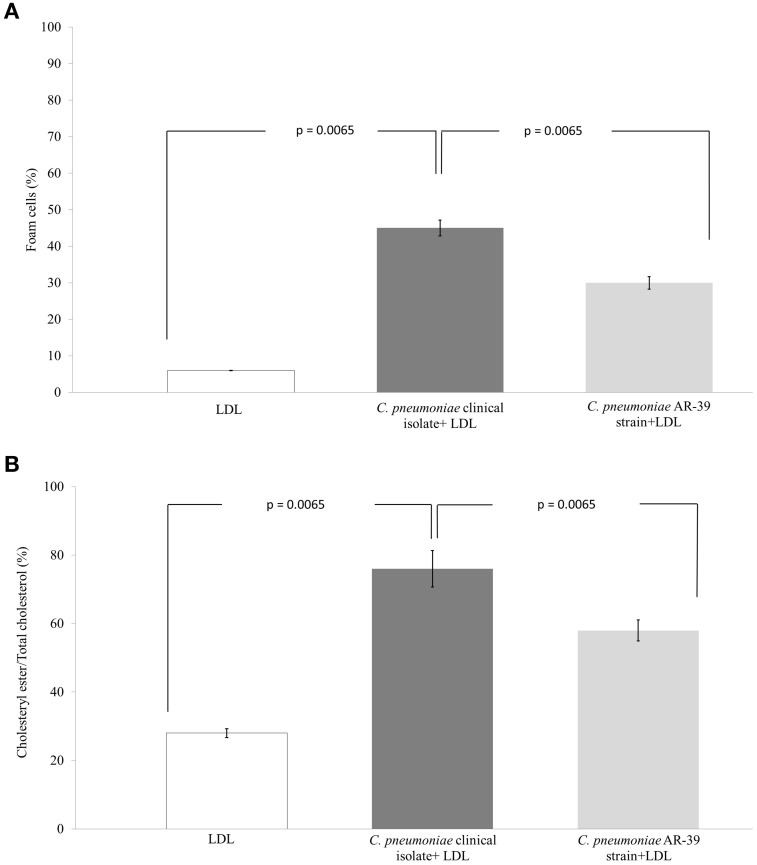
**Comparison of the atherogenic properties between *C. pneumoniae* clinical isolate and AR-39 strain**. The **(A)** foam cell formation and **(B)** the ratio of intracellular cholesteryl ester to total cholesterol in macrophages infected with *C. pneumoniae* clinical isolate and AR-39 strain, in the presence or absence of LDL.

Compared to uninfected and LDL-treated macrophages, *C. pneumoniae* clinical isolate significantly increased the number of foam cells (6 vs. 45%; *P* = 0.0065). Moreover, a significant difference in the number of foam cells between the *C. pneumoniae* clinical isolate (45%) and the AR-39 strain (30%; *P* = 0.0065) was observed (Figures 5, 6A). Lastly, *C. pneumoniae* clinical isolate significantly increased the ratio of intracellular cholesteryl ester to total cholesterol as compared to uninfected LDL-treated macrophages (76 vs. 28%; *P* = 0.0065). A significant difference in the ratio of intracellular cholesteryl ester to total cholesterol between the *C. pneumoniae* clinical isolate (76%) and the AR-39 strain (58%; *P* = 0.0065) was also found (Figure 6B).

### Cytokine levels

IL-1β and IL-6 concentrations were significantly higher in the diseased site (IL-1β: 2.1 ± 0.3 pg/μL; IL-6: 0.6 ± 0.08 pg/μL) than in the healthy periodontal site (IL-1β: 0.51 ± 0.08 pg/μL; IL-6: 0.01 ± 0.004 pg/μL; IL-1β, *P* = 0.0009; IL-6, *P* < 0.0001).

## Discussion

The main result of our study is the isolation, for the first time, of *C. pneumoniae* from the gingival crevicular fluid of a patient with chronic periodontitis, suggesting that this microorganism may harbor inside the oral cavity, acting as a reservoir of the infection. Indeed, other intracellular microorganisms have been detected in the gingival crevicular fluid, supporting the hypothesis that this body fluid could be the intraoral source of the pathogens (Maticic et al., 2001; Suzuki et al., 2005). This may be explained by the characteristics of the gingival crevicular fluid, an inflammatory exudate containing substances from the host, as well as from supra- and sub-gingival located bacteria. Inflammatory and immune cells that have infiltrated into the periodontal tissues are also found in the gingival crevicular fluid together with markers of inflammation, including cytokines and interleukins (Rahnama et al., 2014; Atram et al., 2015).

The significant genomic copy number of *C. pneumoniae*, detected in the gingival crevicular fluid, excludes the transient oral colonization, and the isolation by culture of chlamydial infectious EBs, together with the evidence of intracellular Chlamydia, confirms the presence of an active infection able to contribute to periodontal tissue damage. Interestingly, the detection of a high number of genomic copies paralleled by a low number of infectious EBs suggests the presence of live *C. pneumoniae* and chlamydial persistent forms that seem to be involved in chronic inflammatory diseases (Hogan et al., 2004; Kern et al., 2009). The low number of *C. pneumoniae* infectious EBs and the high amount of chlamydial DNA may also be due to the presence of dead bacteria.

The *C. pneumoniae* strain that we successfully isolated came from a patient who was free, at the time of the specimen collection, from any documented acute or chronic respiratory and cardiovascular disease. However, we cannot rule out the presence of asymptomatic atherosclerotic plaques, such as those of early atherosclerosis, since the patient was at increased risk for cardiovascular diseases. In fact, the estimated 10-year cardiovascular risk according to the Framingham Heart Study was 42%, whereas the normal 10-year risk was 15.1% (D'Agostino et al., 2008).

We investigated the molecular and phenotypical characteristics of the *C. pneumoniae* clinical isolate and we compared them to the AR-39 strain, also known as TWAR as it was isolated in Taiwan in a patient with pharyngitis (Kuo et al., 1995) and whose association with atherosclerosis has been well documented (Watson and Alp, 2008; Campbell and Rosenfeld, 2015). In our study, a 99% similarity was found in the sequence of the VD4 *ompA* gene between *C. pneumoniae* clinical isolate and the AR-39 strain. A high molecular similarity between the two strains is not surprising, since the genomic data from all the sequenced *C. pneumoniae* strains showed a marked genetic conservation (Rattei et al., 2007).

Notably, the phenotypical characteristics of the two strains were also similar. In fact, a comparable growth kinetic in epithelial cells of the *C. pneumoniae* clinical isolate as compared to the AR-39 strain was observed, as evidenced by the infectious progeny and by the number of genomic copies. The similarity of the phenotypical characteristics between the two strains resulted also from a comparable size and morphology of chlamydial inclusions.

Further analysis of the phenotypical features of the *C. pneumoniae* clinical isolate revealed, similarly to the AR-39 strain, its ability to survive and mainly generate infectious EBs within macrophages. The production of infectious EBs in macrophages is of great pathophysiological significance, since it supports the potential ability of *C. pneumoniae* isolate to systematically disseminate through monocytes-macrophages and translocate to the vascular tissue, contributing to the atherosclerotic process. In this regard, several studies have provided the evidence that *C. pneumoniae* disseminates through the PBMCs to the vasculature and to other extra-pulmonary sites (Gieffers et al., 2004; Little et al., 2005; Rupp et al., 2005; Sessa et al., 2007; Watson and Alp, 2008; Di Pietro et al., 2013c).

In our study, *C. pneumoniae* DNA was not detected in the PBMCs of the patient with chronic periodontitis. However, we cannot rule out the potential dissemination of *C. pneumoniae* clinical strain from the oral cavity to the vasculature. Indeed, the low amount of chlamydial infectious EBs isolated from the gingival crevicular fluid may not be enough to allow the translocation of *C. pneumoniae* into PBMCs. The lack of chlamydial DNA in PBMCs may also be due to the putative presence of chlamydial persistent forms in the gingival crevicular fluid, that are not-infectious and, thus, unable to translocate into the systemic circulation. Furthermore, as recently suggested by Zafiratos et al. (2015), only chlamydial antigens may be carried by PBMCs to the vasculature where they become targets of attack by antigen-specific CD8+ T cells, exacerbating the atherosclerotic process. Therefore, monitoring the patient over the time will be needed to demonstrate *C. pneumoniae* dissemination.

Of particular clinical relevance is the foam cell formation following the infection of macrophages, suggesting the potential atherogenic features of *C. pneumoniae* clinical strain. The enhanced foam cell accumulation is considered a key event in the initiation of atherosclerotic plaque development. In this regard, *C. pneumoniae* infection of macrophages has been shown to trigger the oxidation and accumulation of LDL through the release of reactive oxygen species, the increased expression of lipoprotein lipase via chlamydial lipopolysaccharide and the dysregulation of receptors involved in cholesterol efflux via nuclear receptor peroxisome proliferator-activated receptor-γ (Di Pietro et al., 2013a; Cheng et al., 2014; Zhao et al., 2014).

The high similarity of *C. pneumoniae* clinical isolate with the AR-39 strain, usually associated to atherosclerosis (Watson and Alp, 2008; Campbell and Rosenfeld, 2015), emphasizes the importance of the isolation of a potential atherogenic *C. pneumoniae* strain from the oral cavity although further investigations are needed to prove this evidence.

In our study, a further interesting finding is the possible involvement of *C. pneumoniae* in the local chronic inflammation, as suggested by the increased levels of IL-1β and IL-6 detected in the gingival crevicular fluid of the patient with chronic periodontitis. Both these cytokines are major players in the periodontal inflammatory process underlying chronic periodontitis (Faizuddin et al., 2003; Ebersole et al., 2014). Thus, we can hypothesize that, upon *C. pneumoniae* infection, fibroblasts, epithelial cells and macrophages, typically present in the periodontium, may secrete pro-inflammatory cytokines involved in the disease progression and the subsequent destruction of the periodontal tissue.

In conclusion, the isolation of a potential atherogenic strain of *C. pneumoniae* from the gingival crevicular fluid of a patient with chronic periodontitis provides the basis to make some compelling hypothesis. First, *C. pneumoniae* may take part to the initiation and/or the development of chronic periodontitis and, perhaps, contribute to the local chronic inflammatory state. Second, *C. pneumoniae*, similarly to other periodontal pathogens, under favorable conditions such as the destruction of the peridontium, that occurs during periodontitis, may systematically disseminate and localize in the vascular wall, increasing the risk for cardiovascular diseases. However, the data presented in our paper must be interpreted with caution until validated by additional studies in order to clarify the involvement of *C. pneumoniae* in chronic periodontitis as a risk factor for cardiovascular diseases.

### Conflict of interest statement

The authors declare that the research was conducted in the absence of any commercial or financial relationships that could be construed as a potential conflict of interest.
